# New York Journal of Medicine

**Published:** 1845-03

**Authors:** 


*New York Journal of Medicine.*—We are gratified to learn, that the editorship of this Journal, formerly so ably managed by the late lamented and highly gifted, Dr. Samuel Forry, has passed into the hands of a gentleman so competent, in every respect, to conduct it, as Dr. Charles A. Lee, professor of materia medica and general pathology, in the Geneva College. Dr. Lee enjoys a justly deserved high reputation as a man of science and as a writer.—*Balt. Ed.*

